# Paleoparasitological analysis of a 15th–16th c. CE latrine from the merchant quarter of Bruges, Belgium: Evidence for local and exotic parasite infections

**DOI:** 10.1017/S0031182024001100

**Published:** 2024-09

**Authors:** Marissa L. Ledger, Maxime Poulain, Koen Deforce

**Affiliations:** 1 Department of Pathology and Molecular Medicine, McMaster University, Hamilton, Canada; 2 Ancient DNA Centre, McMaster University, Hamilton, Canada; 3 Department of Archaeology, Ghent University, Ghent, Belgium; 4 Royal Belgian Institute of Natural Sciences, Brussels, Belgium

**Keywords:** archeoparasitology, latrine, merchant, paleoparasitology, schistosomiasis, slave trade

## Abstract

Paleoparasitological studies have made important contributions to our understanding of the past epidemiology of parasites, infection in past populations and lifestyle in the past. In some cases, these ancient parasites can also provide evidence for long distance travel or migration of people in the past. Three sediment samples from a 15th–16th c. CE latrine from the Spanish nation house in Bruges, Belgium were analysed for preserved helminth eggs using microscopy. Bruges was a major trading centre in medieval Europe, thus it was home to a large merchant population with extensive trading networks. Paleoparasitological analysis revealed a preserved parasite egg from *Schistosoma mansoni*, which causes intestinal schistosomiasis. Roundworm, whipworm, liver fluke and *Taenia* tapeworm eggs were also found in the latrine which is consistent with parasites previously found in the local population in the medieval period. These new data provide direct evidence for the movement of *S. mansoni* outside of its endemic area. Today the vast majority of *S. mansoni* infections occur in Sub-Saharan Africa, with additional endemic areas in the Arabian peninsula and South America. The introduction of *S. mansoni* into South America is proposed to have occurred relatively recently in human history, as the result of forced movement of people from Africa to the Americas with the Atlantic slave trade. Thus, this infection may have occurred in a merchant who acquired the parasite during trade voyages to Africa or in an individual living in Africa who migrated to Bruges.

## Graphical abstract

**Figure:**
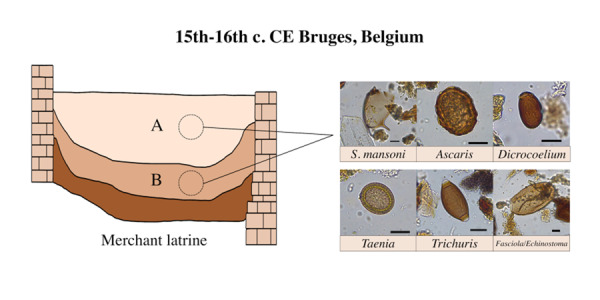

## Introduction

Paleoparasitology, the study of parasites in the past using archaeological materials, has contributed to epidemiological studies revealing parasite infections throughout human history (Le Bailly *et al.,* 2021; Mitchell, 2023). These studies provide data used to understand the interaction between humans and human-infecting parasites over a long time period. Human migration has been linked to the spread of numerous infectious diseases including parasites in prehistoric, historic and modern times. Genomic studies are also an important contributor to elucidating the spread and evolution of these diseases. However, genomic studies using contemporary pathogen genomes can miss the smaller scale movements of infectious diseases that happen on a regular basis. Here we present early direct evidence for the movement of *Schistosoma mansoni* outside of endemic regions with the recovery of parasite eggs from a 15th–16th c. CE latrine in Bruges, Belgium. As a major medieval trading centre with known connections to Africa and early links to the Atlantic slave trade this represents some of the first direct evidence for movement of *S. mansoni* possibly due to migration or travel from Africa to northwestern Europe.

Schistosomiasis affects at least 250 million people worldwide and is classified by the WHO as a neglected tropical disease (Colley *et al.,* 2014; World Health Organization, 2022). Acute and chronic infections are caused by flukes (trematodes) from the genus *Schistosoma,* which live in the blood vessels of infected individuals. There are six species that can cause human infection including *S. mansoni*, *S. haematobium*, *S. japonicum*, *S. intercalatum*, *S. guineensis* and *S. mekongi*. The most common species infecting humans today are *S. haematobium* which causes urogenital schistosomiasis; and *S. mansoni* and *S. japonicum* which cause intestinal schistosomiasis (Ponzo *et al.,* 2024). *Schistosoma* species are geographically restricted based on their snail intermediate hosts. *S. haematobium* and S. *mansoni* are found in Africa and the Middle East (Colley *et al.,* 2014). *S. mansoni* is also endemic in South America and the Caribbean (Gryseels *et al.,* 2006). The remaining species have a more restricted distribution with *S. mekongi* found in the Mekong River Basin and *S. intercalatum* and *S. guineensis* found in West and Central Africa (Bustinduy *et al.,* 2022). Despite this relatively restricted worldwide distribution, *S. mansoni* has effectively spread outside of its previous endemic regions into South America. Modern phylogenetic studies have provided evidence for a relatively recent introduction of *S. mansoni* into South America from Africa, with the proposed timing of this introduction coinciding with the Atlantic slave trade in the 16th c. CE (Crellen *et al.,* 2016; Platt *et al.,* 2022).

Bruges was an important commercial hub in the medieval period, strategically situated at the intersection between northern trade routes of the Hanseatic League and southern trade routes in the Mediterranean, stretching further into Africa and Asia. Its economic success was built on the import and export of both bulk and luxury goods, from cloth to paintings, spurring the development of early commerce and banking systems from the 12th c. until the 16th c. when the economic centre shifted to Antwerp (Brown and Dumolyn, 2018). Bruges was known to host various groups of foreign merchants, coming from Portugal, Spain and Italy among other places. These merchants were concentrated in the commercial heart of the city, where some even established their very own ‘nation house’. These nation houses functioned as an administrative seat and meeting place, used for keeping records, hosting dinners and consular affairs (Dumolyn *et al.,* 2018). Latrine contents from the Spanish nation house were used to understand parasite infection in this medieval merchant community. Previous paleoparasitological analysis of sediments from medieval period sites in Belgium provide a background for the epidemiology of locally transmitted parasites (da Rocha *et al.,* 2006; Rácz *et al.,* 2015; Graff *et al.,* 2020; Rabinow *et al.,* 2023; Wang *et al.,* 2024). Through analysis of the merchant latrine this study aimed to investigate travel or migration related infections that may have been occurring in medieval Bruges, Belgium.

## Materials and methods

**Figure 1.**
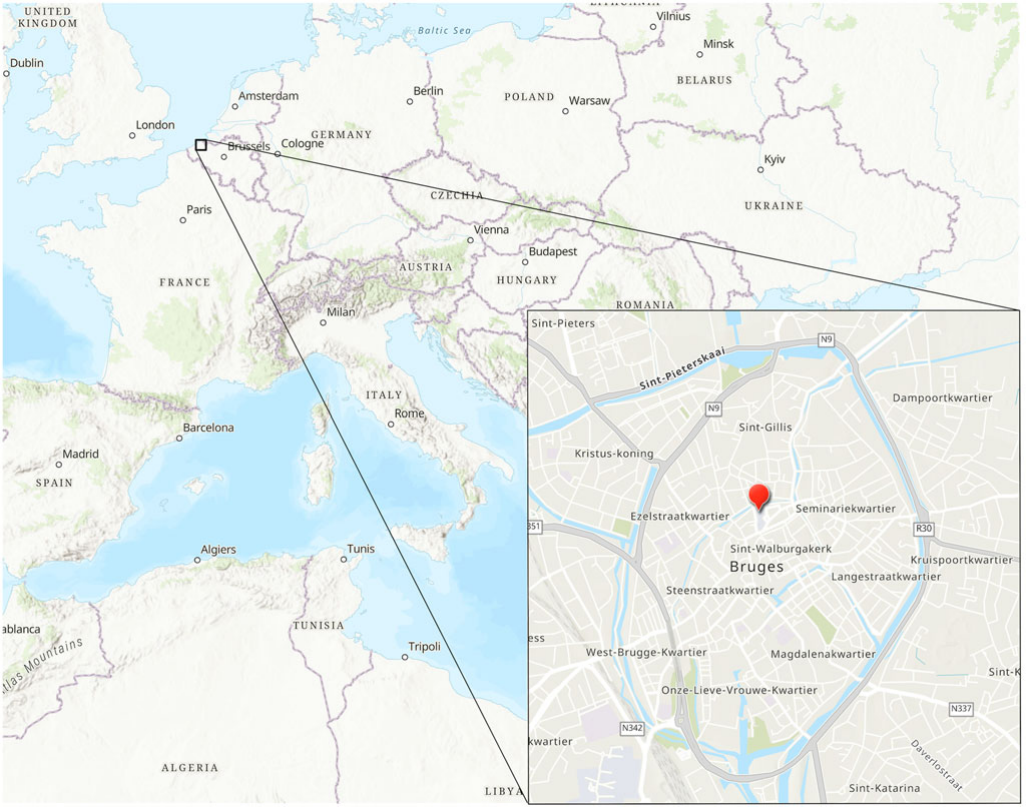
Map showing the location of the latrine (red pin) associated with the Spanish nation house in Bruges. Image credit: Marissa Ledger.

**Figure 2.**
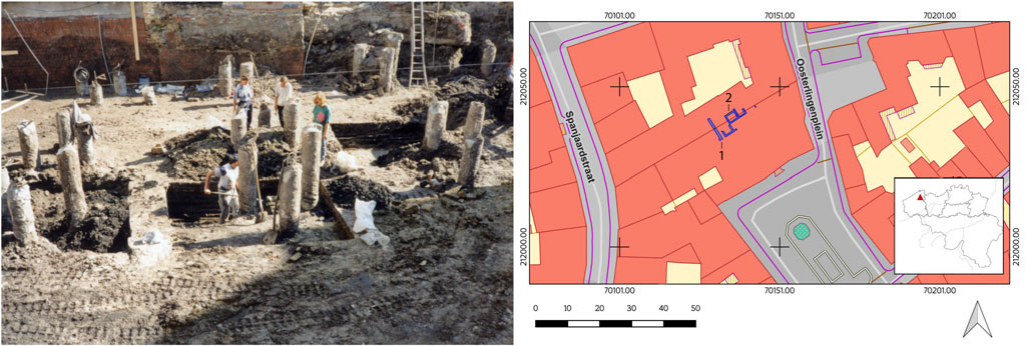
Photo showing excavation site with studied latrine in the centre of the picture (left). Image credit: AZ Sint-Jan, Campus Sint-Franciscus Xaverius. Plan of the excavated structures (right); back wall of the Spanish nation house (1) and studied latrine (2). Image credit: Flanders Heritage Agency.

In 1996 during excavations at the modern day site of the Sint-Franciscus Xaverius hospital (Fig. 1), a latrine was uncovered in the backyard of a late medieval building (De Witte and Hillewaert, 1997) (Fig. 2). The latrine consisted of multiple layers of fill identified during excavation: the lower layer (Layer B) consists of fecal material and smaller organic remains, while the top layer (Layer A) represents the final fill and abandonment of the cesspit. Samples were collected during the 1996 excavations of the latrine and stored at the Bruges Archaeological City Service (now Raakvlak). Three samples were used for this analysis; 2 from Layer B and 1 from Layer A.

Based on a combination of recovered artefacts and radiocarbon dates, both layers could be dated to the 15th through early to mid-16th c. CE. Two cherry stones and a sheep femur from within the fill of the latrine were used to radiocarbon date its use. From the lower layer (Layer B) a cherry stone provided a calibrated date between 1420–1490 CE (95.4% probability, RICH-34345: 436 ± 27 uncal BP), and a sheep femur 1456–1526/1557–1632 CE (RICH-33877: 366 ± 24 uncal BP). A cherry stone from the upper layer (Layer A) provided a calibrated date of 1450–1530/1540–1640 CE (95.4% probability, RICH-34344: 352 ± 26 uncal BP). The youngest artefact in the assemblage, a coin minted in the 1540–1550s, provides a likely date for the abandonment of the latrine.

Historical records provide some information about the individuals who owned and occupied the property in this time period. In 1440, the house is listed as one of the properties rented out by the wealthy butcher and politician Michiel van Theimseke. After a series of transactions, the building came into the hands of the Spanish wool merchant Gómez de Soria in the 1480s. In turn, he decided to sell the property to the city of Bruges in 1494, who subsequently gifted it to the Spanish community to serve as their nation house. Thus, there is an attested Spanish presence at the property from the late 15th c. CE onwards, but Spanish or Italian individuals may possibly have lived at or visited the property earlier, as there was a marked concentration of Mediterranean merchants in this quarter of the city since the 1300s (Marechal, 1953).

Samples were processed in clean rooms following previously published methods within the field of paleoparasitology (Anastasiou and Mitchell, 2013; Ledger *et al.,* 2021). A 0.2 g subsample was disaggregated in 0.5% trisodium phosphate (Callen and Cameron, 1960). As the sediment from the latrine was heavily mineralized, disaggregation required 96 h of soaking in trisodium phosphate with intermittent vortexing. Once all material was disaggregated, samples were microsieved using a stack of sieves with mesh sizes of 300, 150 and 20 *μ*m. The material from on top of the 20 *μ*m sieve was collected and centrifuged at 3100 ***g*** for 5 min. The supernatant was removed and the pellet was mixed with glycerol and placed on microscope slides. Slides were viewed under a light microscope at 400 ×  magnification. The entire 0.2 ***g*** subsample from all three samples was analysed. Parasite eggs were identified using typical morphology and size. The first 100 eggs of each taxa were measured. All eggs were counted, and the total number of eggs of each taxa was multiplied by 5 to get an approximate value of egg concentration in eggs per gram (epg) for all 3 samples.

## Results

**Figure 3.**
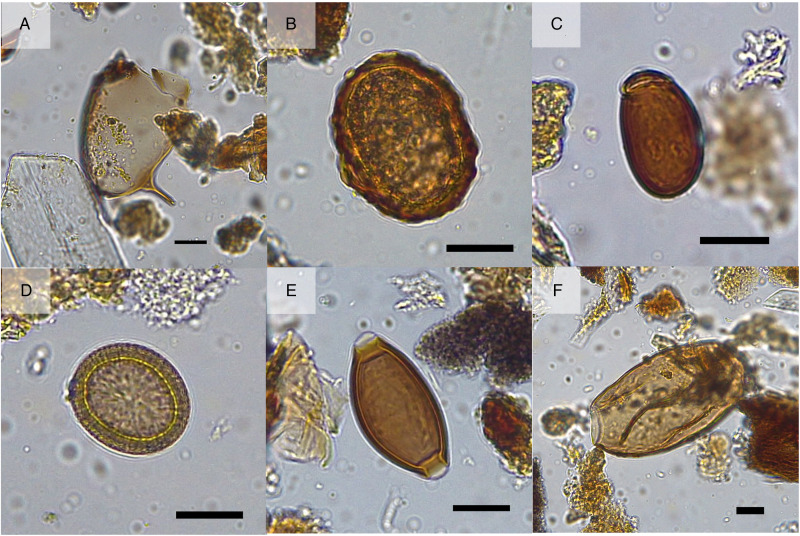
Parasite eggs recovered from the Spanish nation house latrine in 15th–16th c. CE Bruges, Belgium. (A) *Schistosoma mansoni*; (B) *Ascaris* sp.; (C) *Dicrocoelium dendriticum*; (D) *Taenia* sp.; (E) *Trichuris* sp.; (F) Trematode egg. Scale bars indicate 20 *μ*m. Image credit: Marissa Ledger.

Eggs from 6 parasite taxa were recovered from the ancient latrine fill (Fig. 3 and Table 1). There was variation in the parasite taxa recovered from the 2 different layers of the latrine. In the lower layer (Layer B), from which 2 separate subsamples were studied, eggs from *Ascaris* sp. (roundworm), *Dicrocoelium dendriticum* (lancet liver fluke), *S. mansoni*, *Taenia* sp. (beef/pork tapeworm) and *Trichuris* sp. (whipworm) were present. Additionally, one trematode egg was identified with morphology consistent with that of *Fasciola hepatica* or *Echinostoma* sp. The final fill of the latrine (Layer A) contained eggs of *Ascaris* sp., *D. dendriticum* and *Trichuris* sp.

**Table 1.** Parasite taxa recovered from each latrine sample

Sample	Species	Total count (Measured)	Concentration (epg)	Avg. length (*μ*m)	s.d. length	Avg. width (*μ*m)	s.d. width
Layer A	*Ascaris* sp.	142 (100)	710	71.5	5.9	54.7	4.9
	*Dicrocoelium dendriticum*	50 (48)	250	41.6	3.8	26.4	2.5
	*Trichuris* sp.	1238 (100)	6190	55.8	3.2	28.7	1.8
	2 polar plugs	(75)		56.8	2.5	28.7	1.9
	1 polar plug	(6)		54.5	1.7	28.5	1.4
	no polar plugs	(20)		52.5	3.3	28.9	1.6
Layer B-1	*Ascaris* sp.	1619 (100)	8095	68.9	5.8	54.6	4.2
	*Dicrocoelium dendriticum*	2 (2)	10	40.0	NA	29.3	NA
	*Taenia* sp.	3 (3)	15	38.4	NA	NA	NA
	Trematode	1 (1)	5	123.0	NA	67.3	NA
	*Trichuris* sp.	6900 (100)	34 500	55.2	2.8	28.7	1.7
	2 polar plugs	(61)		56.1	2.6	28.8	1.9
	1 polar plug	(12)		54.9	2.2	28.6	1.1
	0 polar plugs	(27)		53.5	2.6	28.8	1.6
	*Schistosoma mansoni*	1 (1)	5	NA	NA	62.4	NA
Layer B-2	*Ascaris* sp.	434 (100)	2170	68.6	6.0	54.3	5.2
	*Dicrocoelium dendriticum*	2 (2)	10	39.2	NA	26.5	NA
	*Trichuris* sp.	1912 (100)	9560	54.8	3.0	28.6	1.6
	2 polar plugs	(72)		55.6	2.6	28.6	1.7
	1 polar plug	(11)		52.2	2.3	28.8	1.6
	0 polar plugs	(17)		53.2	3.8	28.7	1.5

Total number of eggs in the 0.2 g subsample are given with the number of eggs measured to produce morphometric data in brackets. Average length and width with standard deviations are reported for the first 100 eggs of each taxa.

The *S. mansoni* egg recovered (Fig. 3A) had a width of 62.4 *μ*m, consistent with the reported modern size range of eggs, which are between 45–70 *μ*m wide and 140–180 *μ*m long (Garcia, 2016). Unfortunately, the eggshell was broken preventing measurement of the length of the egg. The characteristic lateral spine was preserved allowing for identification to the species level as *S. mansoni* (Garcia, 2016).

*Ascaris* was not identified to the species level given the inability to distinguish between *A. suum* and *A. lumbricoides* (Waindok *et al.,* 2022). While *A. lumbricoides* is the predominant human-infecting species, *A. suum* can occasionally cause infection in humans (Alves *et al.,* 2016; Easton *et al.,* 2020). The eggs were very well preserved as evidenced by the majority still having their mamillated coat (Fig. 3B). *Taenia* sp. eggs were identified based on the typical size and radial striations of the shell. The eggs of *Taenia* and *Echinococcus* cannot be differentiated based on egg morphology alone (Alvarez Rojas *et al.,* 2018; Saelens *et al.,* 2022). However, given their presence in a human latrine these eggs most likely belong to *Taenia* sp. as humans are not a definitive host of *Echinococcus*. This means that while humans can be infected with *Echinococcus* they are not the host of the adult worm, thus eggs are not released in human feces during infection. *Trichuris* eggs were identified based on their typical morphology. Differentiating between species of *Trichuris* using egg morphology can be challenging and is typically done based on size. *T. trichiura* (human whipworm) and *T. suis* (pig whipworm) have overlapping size ranges thus differentiation of these two species is not always possible. The mean length and width of eggs recovered from the latrine is typical for *T. trichiura* (with reported range 49.5–65 by 22.5–30 *μ*m) however, it also falls within the overlapping size range of *T. suis* (46.6–71.2 by 25–37.5 *μ*m) (Beer, 1976). Given that the material studied was from a human latrine these eggs are most likely *T. trichiura*, however, we cannot definitively determine the species based on morphology. One trematode egg was identified based on its morphology, and its size is consistent with that of either *F. hepatica* or *Echinostoma* sp. The typical size range of *F. hepatica* is 130–150 *μ*m by 63–90 *μ*m while *Echinostoma* has a similar appearance but is smaller at 66–149 *μ*m by 43–90 *μ*m (Chai, 2009; Garcia, 2016). The egg identified was 123 *μ*m by 67.3 *μ*m. This egg clearly fits within the size range of *Echinostoma* sp., a parasite that has been previously recovered from medieval Belgium (Rabinow *et al.,* 2023). Of note this egg was missing its operculum thus the measured length does not include the operculum. Even with a preserved operculum it would likely fall just outside or on the low end of the most commonly reported size range for *F. hepatica*. However, there is variability in *F. hepatica* egg sizes in the literature with reports of egg lengths less than 130 *μ*m and even as low as 100 *μ*m (Valero *et al.,* 2009; Qureshi *et al.,* 2019; Loginova *et al.,* 2024; Rabinow *et al.,* 2024). Based on common size ranges reported in human infections, this egg is most likely from *Echinostoma* sp. but it is possible that it is a small *F. hepatica* egg.

The concentration of eggs in the samples studied varied between taxa and between the layers of the latrine (see Table 1). The taxon found in the highest concentration within the latrine was *Trichuris* sp. Concentration of *Trichuris* eggs varied between 34, 500 eggs per gram (epg) in the earlier fill of the latrine (Layer B) to 6,190 epg in the later fill (Layer A). *Ascaris* sp. was the second most numerous with 8,095 epg in the earlier fill and 710 epg in the later fill. In the earlier fill, *D. dendriticum*, and *Taenia* sp. were found between 5 and 15 epg. The only parasite found alongside *Ascaris* and *Trichuris* in the later fill was *D. dendriticum* which was found at a higher concentration (250 epg) compared to the earlier fill which had a more diverse parasite distribution.

## Discussion

The parasite taxa recovered from the Spanish nation house latrine provide important evidence for parasite infection in the mercantile community in medieval Bruges. Parasites recovered that could be locally transmitted include *Ascaris*, liver flukes, *Taenia* and *Trichuris*. *Ascaris* and *Trichuris* were found in very high concentrations in comparison to other taxa which may indicate how common infection with these species was. The presence of *S. mansoni* in the latrine is notable as it has not been recovered from other medieval latrines studied in Belgium unlike the other taxa. In fact, to our knowledge only a single *S. mansoni* egg has been found in medieval Europe outside of its endemic area—from a latrine in France (Bouchet *et al.,* 2002).

In relatively few but influential studies in paleoparasitology, there is clear evidence for migration of parasites with humans (Le Bailly and Bouchet, 2010; Araújo *et al.,* 2014; Araújo *et al.,* 2015; Yeh *et al.,* 2016). The detection of schistosomiasis in the Spanish nation house latrine provides direct evidence for human movement due to its restricted endemic area. Schistosomiasis is endemic in tropical and subtropical regions with infections occurring in Africa, the Arabian peninsula and South America. Specifically, 90% of infections occur in Sub-Saharan Africa with additional areas of endemicity along the Nile river in Egypt and Sudan, northeastern Brazil, Suriname, Venezuela, the Caribbean and the Arabian peninsula (Gryseels *et al.,* 2006). Most cases of *S. mansoni* in Africa occur in Tanzania, Kenya, the Democratic Republic of Congo, Liberia and Ghana (Aula *et al.,* 2021). The geographic distribution of *S. mansoni* is limited by climate and the distribution of its necessary intermediate host, *Biomphalaria* snails (Colley *et al.,* 2014). *S. mansoni* adult worms are found in the mesenteric veins of the bowel in humans where they reproduce and release eggs in the feces. If these eggs contaminate freshwater, in appropriate environmental conditions, they hatch and release miracidia which penetrate snails within which they further develop. Cercariae are then released into water where they can penetrate the skin of humans they come into contact with, completing the life cycle of the parasite (Garcia, 2016; Bustinduy *et al.,* 2022). Eggs can also remain in the intestines or liver where they cause a granulomatous reaction and are responsible for many of the chronic effects of schistosomiasis including abdominal pain, hepatosplenic disease with periportal fibrosis and eventual portal hypertension (Colley *et al.,* 2014). The average lifespan of adult worms is 3–5 years but has been reported to be up to 18 years (Bustinduy *et al.,* 2022), thus they would be able to survive in a human host for the duration of long distance travel in the past.

One study looking at *S. mansoni* infection in contemporary European travellers and migrants showed that 96% of infections occurred in those coming from Africa, and more specifically 36% were acquired in West Africa, 28% in Central Africa and 28% in East Africa (Lingscheid *et al.,* 2017). Thus, if we assume that *S. mansoni* distribution in the 15th–16th c. CE was similar to today, this imported case of schistosomiasis was most likely acquired in Africa and transported to Bruges with the movement of a traveller such as a merchant living in the Spanish nation house or a migrant from Africa. The propensity for *S. mansoni* to be imported with infected travellers and migrants is supported by modern tropical medicine literature providing ample evidence for the detection of this parasite in non-endemic regions of Europe (Grobusch *et al.,* 2003; Amorosa *et al.,* 2005; Aerssens *et al.,* 2011; Lingscheid *et al.,* 2017; Wallemacq *et al.,* 2024). Outbreaks of schistosomiasis have occurred in Europe in recent years and these are predicted to increase as climate change allows for expansion of snail populations into Europe (Kincaid-Smith *et al.,* 2017; van der Deure *et al.,* 2024). However, to date all of these outbreaks have been caused by *S. haematobium* or *S. haematobium*/*S. bovis* hybrids (Steiner *et al.,* 2013; Boissier *et al.,* 2016; Gabrielli and Garba Djirmay, 2023). One reason outbreaks have been limited to *S. haematobium* and hybrid schistosomes is the presence of suitable snail intermediate hosts for these species. Recently the snail intermediate host of *S. mansoni* has been detected in Romania (Majoros *et al.,* 2008), exemplifying the ability of snails to expand their endemic ranges. While there has not yet been evidence for local transmission of *S. mansoni* in the region, the expansion of snail populations could allow for associated expansion of *S. mansoni* to new geographic regions.

Schistosomiasis is an important contributor to parasite burden in modern populations. It is the third leading cause of disability-adjusted life years (DALYs) amongst the neglected tropical diseases (Hotez *et al.,* 2014), though the burden of disease has been argued to be much higher (King *et al.,* 2005; King, 2010). There are an estimated 780 million people at risk of infection and 250 million people infected (World Health Organization, 2022; Díaz *et al.,* 2023). Despite this high prevalence in modern populations, there is little archaeological evidence for schistosomiasis in the human past. Thus, finding *S. mansoni* in this 15th–16th c. CE latrine in Bruges, Belgium contributes to our understanding of the epidemiological history of this important human pathogen.

*S. mansoni* infection in archaeological settings has primarily been identified in Egypt and Nubia (Mitchell, 2024), a known endemic area today. *S. mansoni* DNA was detected in liver tissue using PCR from the mummy Nekht-Ankh found in Rifeh, Central Egypt dating to c. 3900 BP (Matheson *et al.,* 2014). In Nubia, *S. mansoni* eggs were recovered from the pelvic soil of skeletons buried in Kerma (2400–1500 BCE), Sai Island (700–330 BCE and 275 BCE–350 CE) and Sedeinga (13th–14th c. CE) (Harter, 2003). Outside of Egypt and Nubia a single *S. mansoni* egg has been found, in a latrine from 15th c. CE Montbéliard, France (Bouchet *et al.,* 2002). Until now, this was the only evidence for *S. mansoni* outside of endemic regions in the past.

There are a few additional studies that may represent *S. mansoni* infections in the past but they are not conclusive. The mummy Nakht-ROM I found in Thebes and dating to about 1200 BCE, was reported to have calcified eggs of *Schistosoma* spp. in the liver and intestines (Hart *et al.,* 1977; Reyman *et al.,* 1977). Where spines were visible, all were described to have terminal spines consistent with *S. haematobium*. However, it was noted that some eggs had no visible spines thus co-infection with *S. mansoni* could not be excluded. Additionally, *S. mansoni* has presumptively been identified using enzyme-linked immunosorbent assay (ELISA) on mummified tissue from Wadi Halfa, Nubia (350–550 CE) and Kulubnarti, Nubia (550–950 CE) (Miller *et al.,* 1992; Hibbs *et al.,* 2011). These ELISAs are reported to be highly specific for *S. mansoni* however, no indication of cross-reactivity with parasite infections aside from *S. haemotobium* are discussed (Deelder *et al.,* 1989; Barsoum *et al.,* 1992). Cross-reactivity of *Schistosoma* serology with other helminths has been reported for many ELISA tests (Hinz *et al.,* 2017), thus, these results should be interpreted with caution especially given their use on unvalidated specimens.

The rich historical record associated with the Spanish nation house uniquely allows us to understand how *S. mansoni* may have been imported to medieval Bruges. The latrine studied was located within a mercantile environment, and at least some of its contents can be associated with a Spanish merchant household. Trading connections between Bruges and Africa are well documented. The flourishing cloth industry caused a high demand for goods such as alum and dyes, while ‘exotic’ luxury products and spices found avid consumers at the Burgundian court and with the city's urban elite. For example, melegueta pepper (*Aframomum melegueta*), also known as grains of paradise, was traded from Guinea and is encountered in late medieval Belgium, including in the latrine studied here (Collet, 1992; De Clercq *et al.,* 2007; Assië, 2022; De Cupere *et al.,* 2022). Gold dust and ivory were also imported from West Africa, and subsequently redistributed to the rest of Europe (Strubbe, 1953; Guérin, 2010, 2017). The Burgundian residence in Bruges even housed a lion (Bonduel, 2022). These West African products and animals arrived in the Mediterranean using the trans-Saharan trade route, and travelled further north over land or on galleys from the 14th c. onwards. Until that time, the western route *via* Sijilmasa and the Atlas Mountains in Morocco had been preferred, after which trading routes shifted east *via* Mali to Egypt (Bonduel, 2022). While the network linking Bruges to Africa was well established, it should be noted that these connections were indirect and mainly mediated by Italian (Genoese or Venetian), Spanish or Portuguese merchants (Bonduel, 2022).

When we further explore connections that these Mediterranean merchants may have had to Africa, the possibility of this infection occurring in an individual connected to early Atlantic slave trade arises. The Atlantic slave trade began in the 1440s when Portuguese traders undertaking voyages to West Africa in search of gold began to capture and enslave local peoples (Ouattara, 2021). In one of these early voyages traders are documented to have raided local communities to bring enslaved West Africans back to Portugal. The Portuguese involvement in the early slave trade was subsequently supported by the Catholic Church in 1456 and the involvement of Spanish kings was later supported by Pope Alexander VI in 1493 (Otele, 2021: 46). It has been estimated that in the late 15th c. about 1000 slaves were brought to Lisbon annually and merchants would further trade them in the Netherlands, Spain and Italy (Debrunner, 1979: 57; Lawrance, 2005; Phillips, 2013). Spice merchants and textile traders were known to be principal vendors of slaves in 15th c. Genoa, Italy, and African slaves were present in prominent households of Italians, Spaniards and Portuguese in the 1400s (Earle and Lowe, 2005; McKee, 2008).

The oldest written source describing an African individual in Bruges dates back to 1440, when, during the Joyous Entry of Burgundian duke Philip the Good, a contingent of Catalan merchants was heralded by a man described to be from Mauritania (Viaene, 1970). Ethiopian Christians, either on a diplomatic mission or pilgrimage, are documented on multiple occasions in the nearby town of Kortrijk in 1412, 1461 and 1463 (Callewier, 2016). Also in subsequent decades, Africans remain present in various capacities in the historical regions now part of Belgium and Europe in general (Earle and Lowe, 2005). For example, Christophle le More, Charles V's bodyguard, is possibly depicted in the famous painting of a black African man by Jan Mostaert (c. 1525–1530, Rijksmuseum Amsterdam, inv. nr. SK-A-4986). However, African individuals will increasingly feature in written sources as slaves.

Finding *S. mansoni* in the Bruges latrine provides direct evidence for the spread of schistosomiasis outside of its endemic area in the past. This infection is most likely due to the well-documented travel of Italian, Spanish or Portuguese merchants to West Africa or Egypt where they could be infected with schistosomiasis, but may also represent the forced migration of African slaves, or free African individuals, visiting the building known as the Spanish nation house with their owner or as part of an economic, diplomatic or religious mission. Though we cannot say for certain who deposited this parasite in the Bruges latrine, it clearly testifies that this early intercontinental migration resulted in the dispersal of pathogens, in this case, *S. mansoni*. Current evidence suggests that *S. mansoni* was introduced to South America in relatively recent history as a result of the Atlantic slave trade (Platt *et al.,* 2022), thus, it is exceedingly unlikely that this infection was acquired in South America.

The other parasites identified in the latrine from the Spanish nation house are consistent with parasites that have been found in other latrines studied from the region (Deforce, 2010, 2017; Rácz *et al.,* 2015; Graff *et al.,* 2020; Rabinow *et al.,* 2023; Wang *et al.,* 2024). Previously, both *Trichuris* and *Ascaris* have been found in a 15th c. latrine from the palace of the dukes of Burgundy in Bruges (Deforce, 2010). Seven latrines dating between the 12th–17th c. CE in Aalst, Belgium were studied and eggs of *Trichuris*, *Ascaris* and liver flukes (*Echinostoma* and *D. dendriticum*) were found consistently throughout this time period (Rabinow *et al.,* 2023). Additionally, 3 latrines from 14th–17th c. CE Brussels, Belgium revealed eggs from *Trichuris*, *Ascaris*, liver flukes (*D. dendriticum* and *F. hepatica*) and *Taenia* tapeworm (Graff *et al.,* 2020). These data from Belgium show that infection with intestinal parasites was widespread and occurred in all types of households, regardless of their socioeconomic status. All of the parasites found in previous studies from the medieval and early modern periods in Belgium were represented in the latrine from the Spanish nation house. This shows that while those using the latrine in the Spanish nation house were uniquely infected with exotic parasites related to trade and travel from Africa they were also infected with parasites that were transmitted locally.

*Ascaris* and *Trichuris* were found in very high concentrations in the Bruges latrine. Infection with these parasites is acquired by ingestion of eggs typically on food and water contaminated with fecal material or contaminated hands (Else *et al.,* 2020). This indicates poor sanitation conditions. The liver flukes found may represent true infection or false parasitism resulting from ingestion of liver of infected animals. True human infections are rare thus the eggs most likely represent false parasitism (Le Bailly and Bouchet, 2010), and the low egg concentration would support this. Finally, the presence of *Taenia* tapeworm indicates consumption of raw or undercooked beef or pork.

Overall the paleoparasitological analysis of this 15th–16th c. CE latrine from the Spanish nation house in Bruges, Belgium provides unique evidence for the transport of *S. mansoni* over long distances in the past. This coincides with the start of the Atlantic slave trade, and may thus represent infection of a merchant with trade connections to Africa or infection of a slave or free African individual living in Bruges. Those living in and using the latrine in the Spanish nation house were also infected with common parasites transmitted locally including *Ascaris* (roundworm), *Trichuris* (whipworm) and *Taenia* (beef/pork) tapeworm. This study provides important evidence for the epidemiological history of schistosomiasis and important smaller scale movements of *S. mansoni* with human migration and travel in the past.

## Data Availability

All data used within this study are presented within the main text.
